# Comparison of the 2-year clinical performances of class II restorations using different restorative materials

**DOI:** 10.1007/s00784-025-06207-6

**Published:** 2025-02-13

**Authors:** Sevim Hançer Sarıca, Soley Arslan, Hacer Balkaya

**Affiliations:** Faculty of Dentistry, Department of Restorative Dentistry, Erciyes University, Kayseri,, Türkiye

**Keywords:** Bulk-fill composite, Class II restoration, Conventional composite resin, High filler flowable composite resin

## Abstract

**Objective:**

This prospective clinical study aimed to evaluate the two-year clinical performance of conventional composite, bulk-fill composite, and high-filler flowable composite in Class II cavities.

**Materials and methods:**

A total of 259 Class II restorations were performed in 110 patients, applying Clearfil Majesty Posterior (Clearfil) as the conventional composite, Filtek One Bulk Fill Restorative (Filtek) as the bulk-fill composite, and G-aenial Universal Injectable (G-aenial) as the high-fill flowable composite. G-Premio Bond, a universal adhesive system, was applied for all composite resin restorations. Restorations were evaluated using FDI criteria after 2 years. Data were analyzed using the Kruskal-Wallis and Friedman tests.

**Results:**

At the end of two years, the group treated with Clearfil has showed a significantly higher surface gloss score compared to the G-aenial and Filtek groups. Additionally, it was seen that the marginal adaptation scores of the Clearfil group were similar to the Filtek group and significantly higher than the G-aenial group. In intra-group evaluations, the contact point scores of the Clearfil group showed a statistically significant increase compared to baseline and one-year follow-up assessments. The marginal adaptation scores of the Clearfil and Filtek groups also exhibited a statistically significant increase compared to baseline and one-year follow-up assessments.

**Conclusions:**

High-filler flowable composite and bulk-fill composite exhibited better clinical properties regarding surface gloss compared to conventional composite. It was observed that the marginal adaptation property of the conventional composite were similar to the bulk-fill composite and lower than the high- filler flowable composite.

**Clinical relevance:**

: The composite resins tested showed similar results in most of the scores evaluated.

## Introduction

Conventional composite resins are widely used today due to their ease of use, superior physical properties, ability to polymerize, availability in a wide range of colors and translucency, biocompatibility, and satisfactory adhesion to the tooth’s hard tissues [1]. Composite resin materials can also be used to prepare more conservative cavities, reinforce the remaining tooth structure, and enable repair [2, 3].

The main difficulties encountered in using direct composite resins are polymerization shrinkage and shrinkage stress, degree of polymerization conversion, and limited depth of cure. These factors may influence clinical performance. Adequate polymerization and using appropriate placement techniques are critical for the optimal clinical performance of composite resin restorations [4]. It has been suggested that incremental layering of composite resin in increments of ≤ 2 mm reduces shrinkage stress, improves the degree of conversion, prevents restoration margin disintegration, and provides adequate esthetics [5]. However, the incremental application of resin composite is time-consuming. It can be challenging whilst restoring more conservative cavities and may increase the risk of contamination. Also, when incrementally layering composites, void formation between increments can take place, resulting in deterioration of the resin material [6].

The challenges with incremental layering have paved the development of bulk-fill composite materials which may be applied in layers of thickness of 4–5 mm, thereby offering the merit of reduced treatment time, lower technical sensitivity, and the potential of reduced volumetric shrinkage stress as well as improved curing depth [7]. Bulk-filling also prevents deterioration of mechanical properties, with a reduced risk of void formation [8].

It is thought that marginal defects observed in composite restorations generally arise from inadequate adaptation of the restorative material to the cavity walls. Flowable composites, developed for this purpose, adapt well to the cavity margins and walls. Initially, flowable composite resins were not recommended for use in high-stress-bearing areas, large cavities, or occlusal surfaces [9]. With new technological developments, flowable composite resins with higher filler content and modified filler particle size have been produced. These materials exhibit a lower modulus of elasticity, improved mechanical properties, and improved wear resistance, allowing them to be used for all cavity classes (I-V) without capping. This flowable composite is injectable and can maintain its shape during placement. It also has a thixotropic viscosity that allows for very good adaptation to cavity walls and edges. They are aesthetic and the ultra-fine barium particles with a homogeneous distribution ensure a long-lasting gloss thanks to their excellent abrasion resistance. In addition, the flexible tip of the syringe allows access to hard-to-reach areas such as the upper molars [9, 10].

The clinical performance of restorations and restorative materials must be evaluated using detailed, objective, and reliable criteria. One of the most commonly used evaluation criteria for this purpose is the FDI (World Dental Federation). The FDI evaluation criteria are divided into four main groups: functional (fracture or retention issues, form and contour, marginal adaptation, occlusion and wear, and proximal contact points), aesthetic (surface luster, surface texture, marginal staining, color match), biological (caries at restoration margins, dental hard tissue defects at the restoration margin, postoperative hypersensitivity and pulpal status) and miscellaneous (patient’s view, assessment of dental restoration on radiographs) The FDI criteria use a scale where 1 indicates “clinically excellent/very good (sufficient)”, 2 represents “clinically good (sufficient)”, 3 corresponds to “clinically satisfactory (sufficient)”, 4 denotes “clinically unsatisfactory (partially insufficient)”, and 5 signifies “clinically poor (entirely insufficient)“ [11].

The clinical properties of newly developed materials, when evaluated through scientific studies, help dentists familiarize themselves with the materials and select the most suitable and effective ones for their treatments. This, in turn, improves treatment outcomes and increases clinical success [12]. While the properties of bulk fill composite Filtek One Bulk Fill Restorative and conventional composite Clearfil Majesty Posterior have been evaluated in various studies, within our knowledge, there is no study comparing the clinical performance of these materials with the newly introduced high-fill flowable composite, G-aenial Universal Injectable.

This study aimed to evaluate the clinical performance of Class II restorations using conventional composite, bulk-fill composite, and high-filler flowable composite resins according to FDI criteria after two years. The null hypothesis of this study is that there will be no significant difference in the two-year clinical performance of the composite resins used.

## Materials and methods

This clinical trial is registered at www.clinicaltrial.gov (NCT06562868). This study was approved by the Erciyes University Health Sciences Research Ethics Committee with protocol number 2021/604.

### Sample size

The sample size for the study was determined by reviewing similar studies. Using G*Power 3.1 (Faul, 2007) software, the Chi-Square (X2) test family, the Goodness of Fit test: Contingency Tables option was selected. To achieve a medium effect size (Cohen w: 0.35) with an alpha of 0.05 and a power of 0.90, a minimum total sample size of 141 was required, with 47 samples per group. The sample size was increased, and a total of 259 teeth were restored in 110 patients (63 females and 47 males). The mean age of the patients was 24.5 ± 2.5 years (ranging from 19 to 50 years).

## Study design and patient selection

**Fig. 1:**
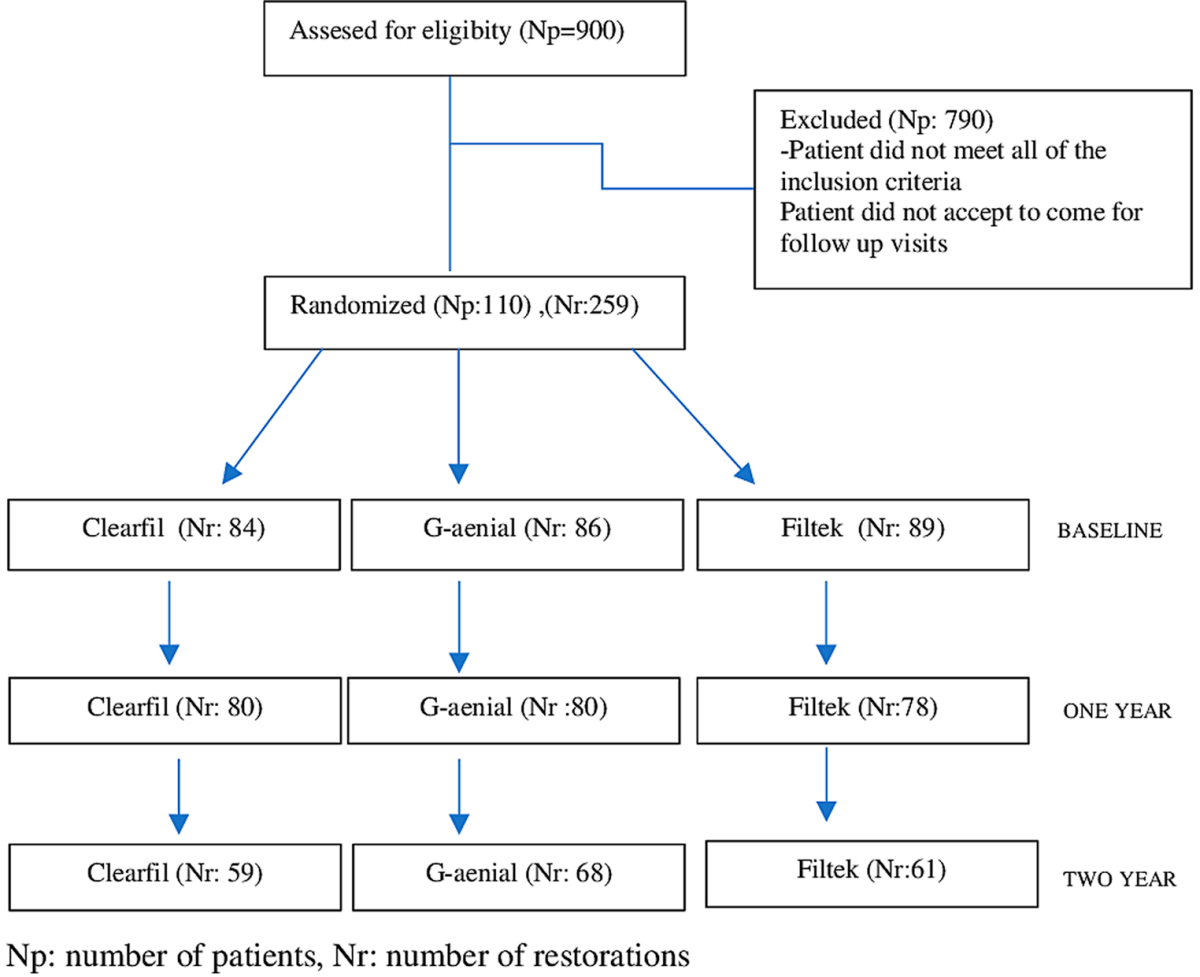
Flowchart of the study

The inclusion and exclusion criteria for the selection of patients for the study are shown in Table 1. In this study, 900 patients were assessed for eligibility for participation, and 790 patients were excluded due to either failing to meet the inclusion criteria or declining to come for follow-up visits. In total, 110 patients who met the inclusion criteria were selected. All volunteers participating in the study were informed about the research, and informed consent was obtained (Fig. 1).

In this study, 110 patients underwent radiographic and intraoral examinations, and 259 teeth requiring restoration were identified. The teeth had Class II caries lesions in the external and middle third of dentin thickness as determined radiographically. All restorations were performed by the same dentist, with extensive clinical experience in restorative dentistry.

**Table 1:** Inclusion/Exclusion criteria

Inclusion Criteria	Exclusion Criteria
-The patient has no systemic disease	-Xerostomia and bruxism
-The patient should be over 18 years of age	-Absence of adjacent and antagonist teeth
-The patient should have good periodontal status	-Extremely poor oral hygiene, severe or chronic periodontitis
-Teeth to be restored should be symptomless and vital	-Pregnant or lactating women
-Teeth to be restored should have proximal contacts on both mesial and distal surfaces and be in occlusion with the antagonist teeth	-Teeth that have any restoration, endodontic treatment, periodontal and periapical pathology.
-Teeth should have class II caries lesions in external and middle 1/3 of dentine thickness radiographically	-The patients who are undergoing orthodontic treatment

## Restorative procedures

The restorative materials were randomly selected using a random number table. Local anesthesia was administered to the teeth to be restored before starting the procedure. Cavity preparations were performed using diamond fissure burs (Diamir srl, Resia, Italy) at high speed with water cooling. Hand instruments and low-speed tungsten carbide burs were used to remove the caries. Conservative cavity design (Class II slot) was used and bevelling was not applied to the cavity walls to avoid unnecessary loss of hard dental tissue. The cavity preparations did not involve any cusps, all the gingival margins included sound enamel, and two surface cavities (MO or DO) were included in this study. The outline shape of the cavity was limited to the removal of caries lesions. Any additional retention was not prepared. The depth of cavities was approximately 4–5 mm from the gingival border of the cavity when the mesial or distal marginal ridge was taken as reference. In our study, after cavity preparation was completed, the cavity was washed and dried.

Since the cavity margins are within the enamel and do not require extensive restoration, cotton rolls, and saliva ejectors were used for isolation [13]. An appropriate matrix system (Tofflemire matrix) and wooden wedges were placed. A 0.2% chlorhexidine gluconate solution was used as a cavity disinfectant. The enamel parts of the teeth were then etched using the selective etch technique with 37% orthophosphoric acid (Eco-Etch, Ivoclar Vivadent, Liechtenstein) for 30 s. After thoroughly washing with water to remove the acid, the tooth was dried with a gentle stream of air. A universal dental adhesive system (G-Premio Bond Universal, GC Corp., Tokyo, Japan) was applied to the cavity using an applicator for 20 s in an active manner according to the manufacturer’s instructions. After being thinned with gentle air for 5 s, the adhesive was polymerized for 10 s using an LED light-curing device (Valo, 1000 mW/cm², Ultradent, USA).

In the first group, Filtek One Bulk Fill Restorative (Filtek) (3 M-ESPE, St. Paul, MN, USA) layers were applied without exceeding 4 mm in thickness. In the second group, Clearfil Majesty Posterior (Clearfil) (Kuraray Medical Inc., Japan) was applied, and in the third group, G-aenial Universal Injectable (G-aenial) (GC Corp., Tokyo, Japan) was applied, with layers not exceeding 2 mm in thickness. The layers were polymerized for 20 s from the occlusal surface using an LED light-curing device. After removing the wedge and matrix, an additional 10 s of light was applied to the surface of the restoration. To remove any excess material and irregularities, finishing was performed under water cooling using fine-grit composite finishing burs (Meisinger Dental Burs, Hager & Meisinger GmbH, Germany) and Sof-Lex (3 M ESPE, St. Paul, MN, USA) discs. Occlusion was checked using articulation paper, and early contact points were removed. The polishing of the restoration was completed using composite polishing rubbers (Nais, Sofia, Bulgaria). Finishing and polishing procedures were performed the same way for all groups.

## Clinical evaluation of the restorations

All restorations were clinically evaluated and scored according to FDI criteria at baseline, after 1 year, and after 2 years by two experienced double-blind dentists using mirrors and probes, as well as bite-wing radiographs and intraoral photographs. In cases where there was disagreement between the dentists in scoring, the final evaluation was based on the joint decision of both dentists. Post-operative sensitivity was scored during the baseline assessment by asking patient-related questions within one week after each restorative procedure. The materials, compositions, and lot numbers are given in Table 2.

**Table 2:** Manufacturer and composition details of the restorative materials used

Materials	Composition
Filtek One Bulk Fill Restorative (3 M-ESPE, St. Paul, MN, USA) LOT NC70264	AFM, AUDMA, UDMA and DDMA. Zirconia/silica and ytterbium trifluoride fillers. 20 nm silica filler, 4–11 nm zirconia filler, zirconia/silica cluster filler, 100 nm ytterbium trifluoride filler. Filler ratio: 76.5% by weight, 58.4% by volume.
Clearfil Majesty Posterior (Kuraray Medical Inc. Japan) LOT 4P0072	BIS-GMA, TEGDMA, hydrophobic aromatic dimethacrylate silane, glass ceramic, micro-filled alumina. Filler ratio: 92% by weight, 82% by volume. Micro-filler: 1.5 μm, Nano-filler: 20 nm.
G-aenial Universal Injectable (GC Corp., Tokyo, Japan) LOT 1903133	DMA, BisMEPP, TEGDMA, pigment, photopolymerization initiators. Filler ratio: 69% by weight, 50% by volume. Silicon dioxide, strontium glass (10–200 nm).
G-Premio BOND (GC Corp., Tokyo, Japan) LOT 2,011,112	Acetone, distilled water, dimethacrylate, 4-META, phosphoric acid ester monomer, silicon dioxide, photopolymerization initiators.

wt%: percentage by weight, vol%: percentage by volume AFM: addition-fragmentation monomer, AUDMA: aromatic urethane dimethacrylate, UDMA: urethane dimethacrylate, DDMA: 1,12-dodecane dimethacrylate, BIS-GMA: bisphenol A-glycidyl methacrylate, TEGDMA: triethylene glycol dimethacrylate, DMA: dimethacrylate, BisMEPP: bisphenol A-glycidyl methacrylate ethoxylated phosphate, 4-META: 4-methylthioethyl methacrylate

### Statistical analysis

The data were analyzed using the IBM SPSS Statistics Standard Concurrent User V 29 (IBM Corp., Armonk, New York, USA) statistical software package. Descriptive statistics were provided as the number of units (n), mean, and standard deviation values. The Kruskal-Wallis analysis was used to compare scores for each material at each measurement time point. Dunn-Bonferroni test was used as the post hoc test in the Kruskal-Wallis analysis. Comparisons based on measurement time points for each material were analyzed using rank-based Friedman analysis. In the Friedman analysis, the Bonferroni correction was applied for pairwise comparisons. A p-value of < 0.05 was considered statistically significant.

## Results

In the study, a total of 110 patients (63 females and 47 males) were evaluated for 259 restorations. The average age of the participating patients was 24.5 ± 2.5 years (ranging from 19 to 50 years). At the end of the first year, 238 restorations (in 86 patients) were assessed, while at the end of the second year, 188 restorations (in 74 patients) were evaluated. Due to relocation and changes in contact information, 24 patients could not be reached at the end of the first year and 36 patients at the end of the second year, resulting in their exclusion from the study.

Scores according to measurement times and intra-group and inter-group score comparisons are given in Tables 3 and 4, respectively.

**Table 3:** Frequencies of score values in materials and measurement times

	Clearfil					G-aenial					Filtek				
	1	2	3	4	5	1	2	3	4	5	1	2	3	4	5
**Surface Gloss**															
Baseline	84					86					89				
1 year	78		2			80					78				
2 years	54		5			67		1			61				
**Surface/Marginal Staining**															
Baseline	84					86					89				
1 year	80					78	2				78				
2 years	58		1			65	3				59	2			
**Color Match**															
Baseline	84					86					89				
1 year	80					79	1				74		4		
2 years	57		2			66	2				57		4		
**Anatomical Form**															
Baseline	84					86					89				
1 year	80					79				1	77				1
2 years	58		1			68					61				
**Fracture**															
Baseline	84					86					89				
1 year	79			1		79				1	78				
2 years	59					68					61				
**Retention Loss**															
Baseline	84					86					89				
1 year	80					79				1	77				1
2 years	59					68					61				
**Marginal Adaptation**															
Baseline	84					86					89				
1 year	76	1	3			78	1			1	74		3		1
2 years	46	3	9	1		65	3				53	1	7		
**Wear**															
Baseline	84					86					89				
1 year	80					80					78				
2 years	59					68					61				
**Contact Point**															
Baseline	84					85		1			89				
1 year	72	3	4	1		76	1	2	1		76	1			1
2 years	50	1	7	1		62	3	2	1		55	5	1		
**Patient Satisfaction**															
Baseline	84					86					89				
1 year	79			1		79			1		77				1
2 years	59					67			1		61				
**Post-op Sensitivity**															
Baseline	84					86					89				
1 year	80					80					78				
2 years	59					68					61				
**Caries/Erosion/Abfraction**															
Baseline	84					86					89				
1 year	80					80					78				
2 years	59					66			1	1	61				
**Tooth Integrity**															
Baseline	54					86					89				
1 year	80					80					78				
2 years	59					68					61				
**Periodontal Response**															
Baseline	84					86					89				
1 year	80					80					78				
2 years	59					68					61				

**Table 4:** Intra-group and inter-group score comparisond according to measurement times

	Clearfil	G-aenial	Filtek	*p* ^‡^
	Mean± Standard Deviation	Mean± Standard Deviation	Mean± Standard Deviation	
**Surface Gloss**				
Baseline	1,00±0,00^*X*^	1,00±0,00	1,00±0,00	-
1 year	1,05±0,31^*X*^	1,00±0,00	1,00±0,00	0,138
2 years	1,17±0,56^*aY*^	1,03±0,24^*b*^	1,00±0,00^*b*^	**0**,**019**
*p* ^†^	**0**,**022**	0,368	-	
**Surface/Marginal Staining**				
Baseline	1,00±0,00	1,00±0,00	1,00±0,00	-
1 year	1,00±0,00	1,03±0,16	1,00±0,00	0,138
2 years	1,03±0,26	1,04±0,21	1,03±0,18	0,700
*p* ^†^	0,368	0,097	0,135	
**Color Match**				
Baseline	1,00±0,00	1,00±0,00	1,00±0,00^*X*^	-
1 year	1,00±0,00	1,01±0,11	1,10±0,44^*Y*^	0,063
2 years	1,07±0,37	1,03±0,17	1,13±0,50^*Y*^	0,531
*p* ^†^	0,135	0,223	**0**,**018**	
**Anatomical Form**				
Baseline	1,00±0,00	1,00±0,00	1,00±0,00	-
1 year	1,00±0,00	1,05±0,45	1,05±0,45	0,601
2 years	1,03±0,26	1,00±0,00	1,00±0,00	0,335
*p* ^†^	0,368	0,999	0,999	
**Fracture**				
Baseline	1,00±0,00	1,00±0,00	1,00±0,00	-
1 year	1,04±0,34	1,05±0,45	1,00±0,00	0,616
2 years	1,00±0,00	1,00±0,00	1,00±0,00	-
*p* ^†^	0,998	0,995	-	
**Retentation Loss**				
Baseline	1,00±0,00	1,00±0,00	1,00±0,00	-
1 year	1,00±0,00	1,05±0,45	1,05±0,45	0,597
2 years	1,00±0,00	1,00±0,00	1,00±0,00	-
*p* ^†^	-	0,992	0,991	
**Marginal Adaptation**				
Baseline	1,00±0,00^*X*^	1,00±0,00	1,00±0,00^*X*^	-
1 year	1,10±0,41^*X*^	1,06±0,46	1,13±0,59^*X*^	0,652
2 years	1,40±0,81^*aY*^	1,04±0,21^*b*^	1,25±0,65^*abY*^	**0**,**010**
*p* ^†^	**<0**,**001**	0,097	**0**,**002**	
**Wear**				
Baseline	1,00±0,00	1,00±0,00	1,00±0,00	-
1 year	1,00±0,00	1,00±0,00	1,00±0,00	-
2 years	1,00±0,00	1,00±0,00	1,00±0,00	-
*p* ^†^	-	-	-	
**Contact Point**				
Baseline	1,02±0,22^*X*^	1,02±0,22	1,00±0,00	0,368
1 year	1,18±0,57^*Y*^	1,10±0,47	1,06±0,46	0,141
2 years	1,33±0,77^*Z*^	1,15±0,53	1,11±0,37	0,266
*p* ^†^	**0**,**001**	0,130	0,062	
**Patient Satisfaction**				
Baseline	1,00±0,00	1,00±0,00	1,00±0,00	-
1 year	1,04±0,34	1,05±0,45	1,05±0,44	-
2 years	1,00±0,00	1,04±0,36	1,00±0,00	0,411
*p* ^†^	0,985	0,368	0,965	
**Post-op Sensitivity**				
Baseline	1,00±0,00	1,00±0,00	1,00±0,00	-
1 year	1,00±0,00	1,00±0,00	1,00±0,00	-
2 years	1,00±0,00	1,00±0,00	1,00±0,00	-
*p* ^†^	-	-	-	
**Caries/Erosion/Abfraction**				
Baseline	1,00±0,00	1,00±0,00	1,00±0,00	-
1 year	1,00±0,00	1,00±0,00	1,00±0,00	-
2 years	1,00±0,00	1,10±0,60	1,00±0,00	0,167
*p* ^†^	-	0,548	-	
**Tooth Integrity**				
Baseline	1,00±0,00	1,00±0,00	1,00±0,00	-
1 year	1,00±0,00	1,00±0,00	1,00±0,00	-
2 years	1,00±0,00	1,00±0,00	1,00±0,00	-
*p* ^†^	-	-	-	
**Periodontal Response**				
Baseline	1,00±0,00	1,00±0,00	1,00±0,00	-
1 year	1,00±0,00	1,00±0,00	1,00±0,00	-
2 years	1,00±0,00	1,00±0,00	1,00±0,00	-
*p*†	-	-	-	

Data are given as mean±standard deviation, p†: Friedman analysis, p‡: Kruskal-Wallis test, superscripts a and b indicate between-group differences. Groups with the same superscripts are not statistically different. Superscripts X, Y, and Z indicate within-group differences. There is no statistical difference between measurements with the same superscripts

While there was no statistically significant difference between the groups at the end of 1 year in terms of the evaluated criteria, at the end of 2 years the surface gloss scores in the Clearfil group were statistically higher compared to both the G-aenial and Filtek groups (*p* < 0.05). In addition, at the end of 2 years, the marginal adaptation scores of Clearfil group were similar to Filtek group and were statistically significantly higher than those of G-aenial group (*p* < 0.05).

In intra-group comparisons, a statistically significant increase was observed in color matching scores at the end of 1 year in the Filtek group compared to the baseline scores (*p* < 0.05), while there was no significant difference between 1 and 2 years. In addition, a statistically significant increase was observed in the marginal adaptation scores of both the Clearfil and Filtek groups after 2 years compared to the baseline and 1 year scores (*p* < 0.05). Additionally, the Clearfil group’s contact point scores after 2 years showed a statistically significant increase compared to both baseline and 1 year scores (*p* < 0.05).

## Discussion

This study aimed to evaluate the two-year clinical performance of Class II cavities restored with conventional composite, bulk-fill composite, and highly filled flowable composite resins according to FDI criteria. Based on the results of this study, the null hypothesis, which predicted that there would be no significant difference in the clinical performance of the composite resins used after two years, was rejected.

In vitro studies are commonly conducted in laboratory settings to examine the mechanical and physical properties of composite resin materials. Although many parameters can be measured using this method, in vitro studies may be insufficient to evaluate the clinical performance of materials, as they cannot fully replicate the dynamics of the oral environment. Clinical studies are the most effective method for assessing the clinical performance of existing materials and comparing the clinical properties of different materials [14]. For this reason, our study was designed as a clinical study.

The success of clinical studies depends on the effectiveness of patient follow-up. However, various obstacles, such as patients’ reluctance to attend appointments, address changes, or health issues, can make regular follow-ups challenging, leading to patient attrition and data loss, particularly in studies requiring long-term follow-up. This situation often results in gaps in the literature regarding long-term follow-up studies. At the beginning of our study, the minimum sample size per group was determined as 47 to achieve a medium effect size with an alpha of 0.05 and a power of 0.90. However, considering the possibility of attrition during follow-up, we increased the initial sample size in each group by approximately 80%. This approach provided flexibility against potential losses, ensuring the reliability and validity of the data despite the reduction in the number of patients at the end of the two years.

Conventional composite resins have been successfully used in the restoration of posterior teeth for many years. However, studies have reported that conventional composite resin restorations exhibit polymerization shrinkage, insufficient resistance to occlusal forces, and deficiencies in marginal adaptation [15]. New technologies, such as bulk-fill composite resins and highly filled flowable composite resins, have addressed these issues, resulting in more successful outcomes [16]. Therefore, in our study, we utilized bulk-fill composite resins and highly filled flowable composite resins, which were developed as alternatives to conventional composite resins.

USPHS and FDI criteria are the most commonly used evaluation standards in clinical studies. Both criteria are easy to apply and interpret, providing information on the acceptability of restorations [17]. However, FDI criteria include more evaluation parameters, allowing for more detailed and precise results [11]. Thus, in our study, FDI evaluation criteria were used to assess the clinical performance of restorations. When the 1-year clinical performances of the composite resins used in our study were evaluated, no significant difference was found between the materials, but a significant difference in tersm of surface gloss and marginal adaptation criteria was observed at 2-year follow up.

Balkaya et al. [18] reported no difference in the clinical performance of materials, according to USPHS criteria, during the 1- and 2-year clinical follow-ups of Class II restorations using a conventional composite resin (Charisma Smart Composite) and a bulk-fill composite resin (Filtek Bulk Fill Posterior Restorative). Similarly, in a study by Çolak et al. [19], bulk-fill composite resin (Tetric EvoCeram Bulk Fill) and nanohybrid composite resin (Tetric EvoCeram) were applied to Class II cavities, and evaluations conducted after one year using USPHS criteria revealed no differences between the materials. Kitasako et al. [20] evaluated the clinical performance of a conventional composite resin (Estelite Sigma Quick) and a highly filled flowable composite resin (G-aenial Universal Flo) in posterior regions over three years using FDI criteria and reported no significant differences between them.

The surface roughness and gloss of composite resins are influenced by various factors, such as the type, size, and quantity of monomers and filler particles constituting the organic matrix [19]. As particle size decreases, the surface becomes smoother and glossier after polishing [20]. For instance, Daud et al. [21] evaluated the effects of different finishing and polishing techniques on the surface roughness of microhybrid and nanofill resin composites. They observed that the initial samples of Filtek Supreme XTE were smoother than those of Filtek Z250. This was attributed to the presence of nano-sized particles averaging 11 nm in Filtek Supreme XTE, whereas Filtek Z250 contained microhybrid particles averaging 0.6 μm in size.

The monomer content of composite resins is important. Bis-GMA-containing composites exhibit greater affinity for water compared to UDMA-containing ones, with water uptake increasing by 3–6%. Adding small amounts of TEGDMA to a Bis-GMA-based resin matrix can further increase water absorption by 0–1%. The hydrophilic properties of Bis-GMA and TEGDMA monomers in the structure of composite resins can soften the matrix through water absorption, leading to the degradation of the composite surface. This can result in the exposure of inorganic fillers and an increase in surface roughness [22]. In a 36-month clinical follow-up study conducted by Yazıcı et al. [23], it was reported that two restorations in the conventional composite resin (Filtek Ultimate) group exhibited slightly rougher surfaces compared to those in the bulk-fill composite resin (Tetric EvoCeram BulkFill) group.

In our study, at the two-year evaluation, it was observed that the surface gloss scores of Clearfil were statistically significantly higher than those of G-aenial and Filtek. The higher surface smoothness and gloss scores of Clearfil may be related to the presence of hydrophilic monomers such as Bis-GMA and TEGDMA, as well as the larger filler particles in this composite compared to other materials. Additionally, this could also be associated with voids remaining during the layered placement of Clearfil [23].

Marginal adaptation is a criterion that evaluates the fit of the restoration edges to the tooth and is considered one of the most critical factors determining the clinical success of restorations [24]. The boundaries between the tooth and the restoration should be smooth and continuous. Disruption of this fit is often associated with the early formation of gaps and the degradation of the tooth, adhesive, and composite surfaces at the bonding interface. The formation of gaps is caused by volumetric changes in resin-based materials due to polymerization shrinkage stress during the polymerization process [25]. Furthermore, although the increased filler content in commonly used high-viscosity composite resins improves the physicomechanical and handling properties of these materials, it also makes it challenging to adapt the restorative material to cavity walls, leading to the formation of interface gaps and increased microleakage [26]. Over time, the wear of composite resin restorations and the hydrolysis of the adhesive interface due to the absorption of water and chemicals by monomers can adversely affect marginal adaptation [27]. Additionally, the adhesion strength between the composite resin and dentin is influenced by various factors, such as the quality of the tooth’s hard tissues, as well as the orientation and diameter of dentinal tubules [28]. The marginal adaptation scores of the Clearfil group were significantly higher than the G-aenial group in the two-year evaluation of our study. On the other hand, the marginal adaptation scores of the Clearfil group were found to be statistically similar to the Filtek group, while they were numerically higher than the Filtek group. Additionally, in our study, significant increases in marginal adaptation scores of both Filtek and Clearfil applied groups were observed during intra-group evaluations.

Clearfil contains a resin matrix with 92% filler weight and 82% filler volume. Its high filler content and the necessity of using a layered placement technique may result in insufficient condensation to the cavity walls and compromised marginal adaptation [29]. Moreover, it contains monomers such as Bis-GMA and TEGDMA. Shrinkage in the material’s volume during polymerization, due to Clearfil’s monomer content, can result in gap formation at the bonding interface, ultimately impairing marginal adaptation. Furthermore, the highwater absorption capacity of these monomers may have contributed to hydrolysis and marginal adaptation issues [30].

Bulk-fill composite resins are restorative materials that hold great promise for restorative dentistry and have demonstrated success in in vitro evaluations. These materials were proposed with the expectation of compensating for the issues associated with conventional composite resins, such as polymerization shrinkage stress. Bulk-fill composites offer modifications in their chemical composition, such as monomeric adjustments and initiator systems, to reduce polymerization shrinkage. While some studies in the literature have reported no significant difference in marginal gap formation and adaptation between conventional and bulk-fill composite resins, others have suggested that bulk-fill materials provide better marginal adaptation [31, 32]. Another study indicated that high-viscosity bulk-fill composite resins may lead to larger marginal gap formation [33].

The good marginal adaptation demonstrated by G-aenial can be attributed to its high thixotropic viscosity. This characteristic allows it to adapt very well to cavity walls and edges, filling gaps, or voids. Additionally, G-aenial’s high physical, chemical, and mechanical properties can be linked to its Full-Coverage Silane Coating (FSC) technology [34].

In anterior teeth, the aesthetic harmony of the restoration with the tooth color is of great importance, while in posterior teeth, it is generally sufficient for the restoration to be close to the tooth color. Color mismatches between the restoration and the tooth may result from the initially selected material color or changes in the material over time [35]. Factors such as the structure of the composite resin, water absorption, polymerization method and duration, finishing and polishing procedures, staining agents, surface properties, patient oral hygiene, and the age of the composite resin have all been reported to influence the color stability of restorations [36].

In our study, evaluations based on the color matching criterion of the materials revealed that, in intra-group comparisons, only Filtek showed statistically significant higher color matching scores at the end of one year compared to the baseline scores. Similar to our findings, a study by Serin-Kalay et al. [37] reported that bulk-fill materials are more sensitive to color changes than conventional composites. A 36-month clinical follow-up study by Loguercio et al. [38] observed minor deviations in color matching in bulk-fill composites due to their increased light transmittance and structural properties. Additionally, an in vitro study by Meena et al. [22] reported that conventional composites (Clearfil Majesty Posterior) exhibited less color change compared to bulk-fill resin composites (SureFil SDR, Ever X, Tetric Evo Ceram). The observed color differences in bulk-fill composites can be associated with improved formulations, such as increased photoinitiator content, the use of an additional photoinitiator type, higher translucency, and the incorporation of different filler types. Moreover, the blue-light absorbing photosensitizers and camphorquinone used as amine initiators in the photoinitiator systems of composite resins may contribute to oxidative color changes [39]. The literature also suggests that composite resins containing metal ions may accelerate hydrolytic degradation mechanisms [40]. The color change observed in the bulk-fill composite resin used in our study is thought to be related to the metal fillers (such as ytterbium and zirconium) in these composites and the structural properties mentioned above.

A good proximal contact is crucial for functional chewing. If the contact is too loose, food impaction, tooth movement, periodontal problems, and caries can occur. On the other hand, excessively tight contact can lead to tooth movement or periodontal tissue trauma when attempting to pass dental floss with excessive force [41]. Dynamic forces are applied to the contact points of restorations during chewing. Although composite materials have undergone significant advancements over time, their resistance to wear and fracture in occlusal contact areas and proximal contact points remains a concern. It is known that the mechanical properties of dental composites depend to a greater extent on the concentration of the filler and the particle size. Composite resins with a higher filler content have a higher elastic modulus and are more susceptible to brittle fracture [42]. Nanometric inorganic fillers added to composite resins, due to their relatively small size and rounded shape, present a high surface area and consequently require a higher amount of silane. These silanized nanofiller particles result in an increase in the mechanical properties of the composite resin. In addition, these rounded particles tend to distribute the mechanical stress more uniformly than irregularly shaped particles, which present sharp angles known as stress concentration areas where cracks can initiate [43]. Additionally, a study evaluating the surface properties of composite resin restorations revealed that all restoration surfaces initially contained excess material that was not visible to the naked eye and could not be clinically detected during occlusion control but was identified microscopically. This excess material, initially present and disappearing for approximately five years, was noted as a significant disadvantage for tooth-colored materials [44].

Loomans et al. [45] conducted an in vitro study to measure the fracture resistance of Class II restorations by applying force to the contact points using Filtek Supreme, Clearfil AP-X, and Clearfil Majesty Posterior. The study revealed that Clearfil Majesty Posterior exhibited lower fracture resistance compared to Clearfil AP-X and Filtek Supreme. They attributed this to Clearfil Majesty Posterior’s significantly higher filler content and elastic modulus compared to other composites, as well as the weaker interaction between its filler particles, silane agent, and resin matrix. In a clinical study by Torres et al. [46], Class II cavities were restored using conventional composite resin (GrandioSO) and highly filled flowable composite resin (GrandioSO Heavy Flow). After a 2-year follow-up, the proximal contact scores of the conventional composite were reported to be higher than those of the highly filled flowable composite.

In our study, intra-group comparisons also demonstrated an increase in contact point scores for Clearfil at the end of two years. The increase in Clearfil’s contact point scores can be attributed to factors such as excess material that is not detectable or removable to the naked eye, its high elastic modulus due to a higher filler content compared to other composite resins used in the study, and its low flexural strength.

While our study provides valuable insights into the clinical performance of composite resins with different clinical application procedures, a two-year period is insufficient to evaluate the long-term clinical success of composite resins and decrease in the number of patients after 2 years, which is a limitation of our study.

## Conclusions

The composite resins tested showed similar results in most of the scores evaluated. Besides that high-filler flowable composite and bulk-fill composite exhibited better clinical performance regarding surface gloss compared to conventional composite. Additionally, it was observed that the marginal adaptation property of the conventional composite were similar to the bulk-fill composite and lower than the high- filler flowable composite.

## Data Availability

No datasets were generated or analysed during the current study.
